# Supplementary material for: A lipid transfer-dependent feedback loop activates ATG9A compartments in autophagy initiation
Source: Nat Commun. 2026 Aug 31;17:10374. doi: 10.1038/s41467-026-77368-5 (PMC13627691; doi:10.1038/s41467-026-77368-5)
Supplement: Supplementary file 1 [file 41467_2026_77368_MOESM1_ESM.pdf]

**Supplementary Fig. 1**

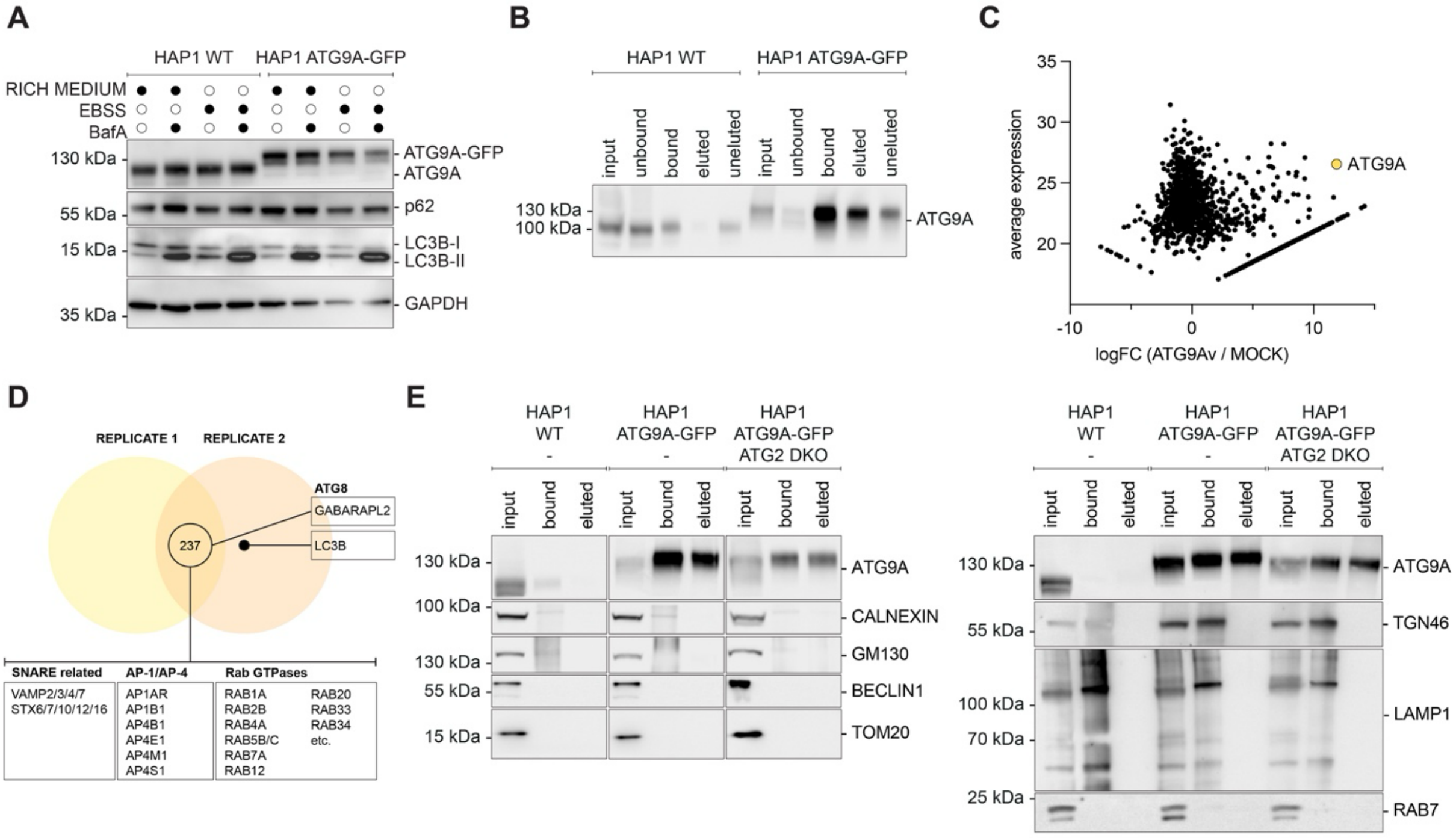


**Supplementary Fig. 1. Isolation and analysis of native ATG9A compartments**

(**A**) Western blot analysis of cell lysates from HAP1 wild-type and HAP1 ATG9A-GFP knock-in cell lines grown in rich medium or EBSS, either untreated or treated with 100 nM Bafilomycin A1 (BafA1) for 2 hours. Both the wild-type and knock-in cells display comparable levels of autophagy flux (LC3B), indicating that the GFP and FLAG tags do not impair ATG9A functionality. (**B**) Western blot analysis of fractions collected during the isolation of native ATG9A compartments. ATG9A is strongly enriched in the final eluate from the HAP1 ATG9A-GFP knock-in cell line, while it is nearly undetectable in the corresponding HAP1 wild-type (WT) control, indicating successful and specific enrichment of ATG9A compartments. (**C**) Volcano plot showing mass spectrometry results. ATG9A (yellow) is significantly enriched in vesicles isolated from HAP1 ATG9A-GFP cells compared to HAP1 wild-type (MOCK) cells, confirming the specificity of the isolation procedure. (**D**) Venn diagram showing the overlap of proteins identified in two independent replicates of ATG9A compartment isolations, highlighting shared proteins among the top 1000 hits. (**E**) Western blot analysis of input (0.5% of sample loaded), bound (2.8% of sample loaded), and eluted fractions (2.8% of sample loaded) from ATG9A vesicle isolations performed using HAP1 wild-type, ATG9A-GFP, and ATG9A-GFP ATG2 DKO cells. Approximately 1.2 × $10^8$ cells were lysed per isolation. ATG9A is enriched in the bound fraction from ATG9A-GFP and ATG9A-GFP ATG2 DKO cells, whereas markers of other membrane compartments, including EEA1, GM130, BECLIN1, TOM20, TGN46, LAMP1, and RAB7, are not co-purified.

**Supplementary Fig. 2**

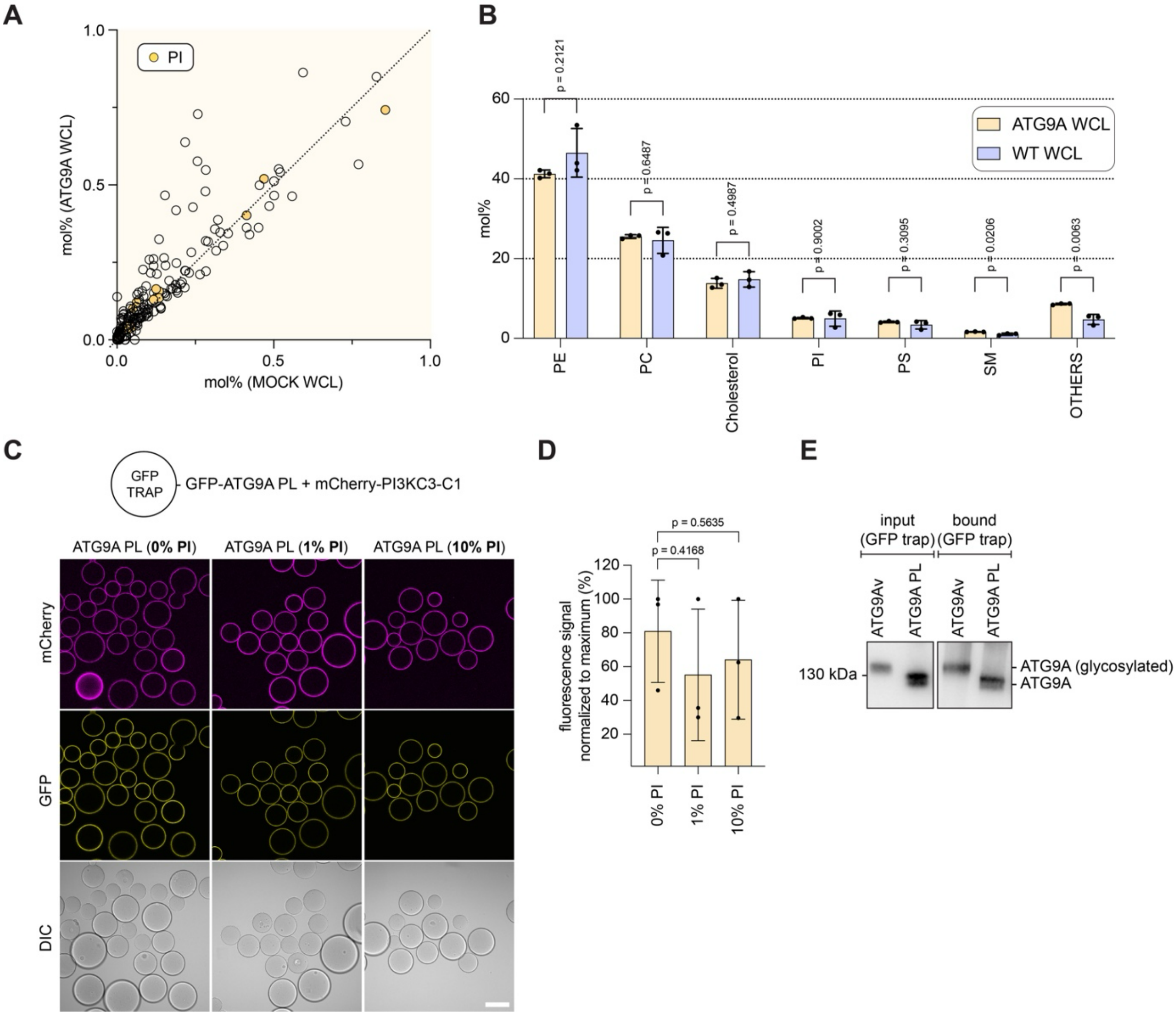


## Supplementary Fig. 2. Analysis of native ATG9A compartments

(**A**) Scatter plot comparing the molar percentage (mol %) of individual lipid species in whole cell lysates of HAP1 wild-type cells (x-axis) and ATG9A-GFP cells (y-axis). Each point represents a lipid species. The clustering along the diagonal indicates a high similarity in lipid composition between the two cell lines. PI species are highlighted in yellow. (**B**) Lipidomic analysis of whole cell lysates (WCL) from HAP1 ATG9A-GFP (yellow) and wild-type (violet) cells. Lipid species were grouped by class, and their molar percentages were summed to yield the total mol% per lipid class shown on the x-axis. CE, Cer, DAG, GlcCer, LacCer, PG, and TAG were summarized under "others". Statistical significance within each class was assessed using an unpaired *t*-test. Bars represent mean ± s.d. (**C**) GFP-Trap beads coated with ATG9A PL were incubated with mCherry-PI3KC3-C1 (30 nM) to assess whether recruitment of PI3KC3-C1 depends on PI. **(D)** Quantification of mCherry fluorescence from panel **C** (*N* = 3; independent experiments). Statistical significance was assessed using an unpaired *t*-test. Bars represent mean ± s.d. mCherry signal (background-corrected) was expressed relative to

the maximum value of each replicate. **(E)** Comparison of ATG9A-GFP levels in native ATG9A-containing vesicles and reconstituted ATG9A PLs. ATG9A-containing vesicles were isolated from HAP1 ATG9A-GFP cells. Reconstituted ATG9A PL were composed of 50% POPE, 20% cholesterol, 27% DOPC, 2% DOPS, and 1% sphingomyelin. For each condition, 300 μL of sample were subjected to GFP-Trap pull-down using 4 μL GFP-Trap bead slurry. Bound fractions were analyzed by Western blot. The higher apparent molecular weight of ATG9A-GFP in vesicle-derived samples is due to its glycosylation in mammalian cells, whereas the lower apparent molecular weight observed in PL samples reflects ATG9A purified from insect cells.

**Supplementary Fig. 3**

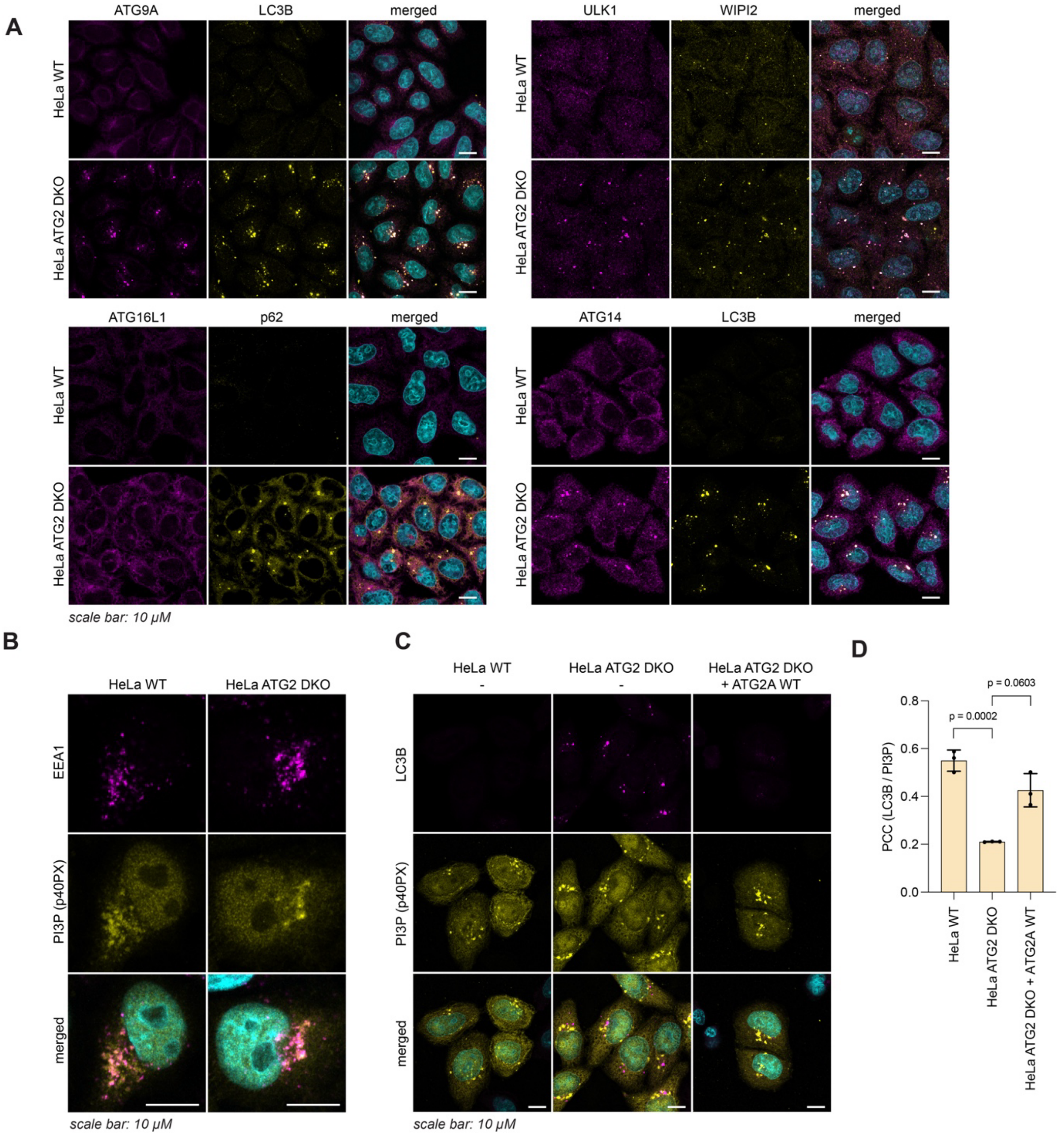


**Supplementary Fig. 3. Foci formation in HeLa ATG2 DKO cells**

(**A**) Immunofluorescence staining of HeLa wild-type and ATG2 DKO cells for autophagy-related proteins: ATG9A, LC3B, ATG16L1, p62, ULK1, WIPI2, and ATG14. ATG2 DKO cells show aberrant accumulation of autophagy markers in distinct foci. (**B**) Immunofluorescence staining of HeLa wild-type and ATG2 DKO p40PX-EGFP (PI3P-binding domain) for analyzing the co-localization of EEA1 and p40PX as a control for specificity of the detector (**C**) Rescue of foci phenotype in ATG2 DKO cells 24 h after transient transfection with a wild-type ATG2A construct. LC3B localization resembles that of HeLa wild-type cells, indicating restoration of

normal trafficking. **(D)** Quantification of co-localization between LC3B and p40PX-EGFP in HeLa WT, ATG2 DKO cells, and ATG2 DKO cells transfected with a wild-type ATG2A construct (shown in **C**), assessed using Pearson’s correlation coefficient (PCC). Statistical significance was determined using an unpaired *t*-test ($N$ = 3; independent experiments; 3 images per condition and replicate). Bars represent mean ± s.d.

**Supplementary Fig. 4**

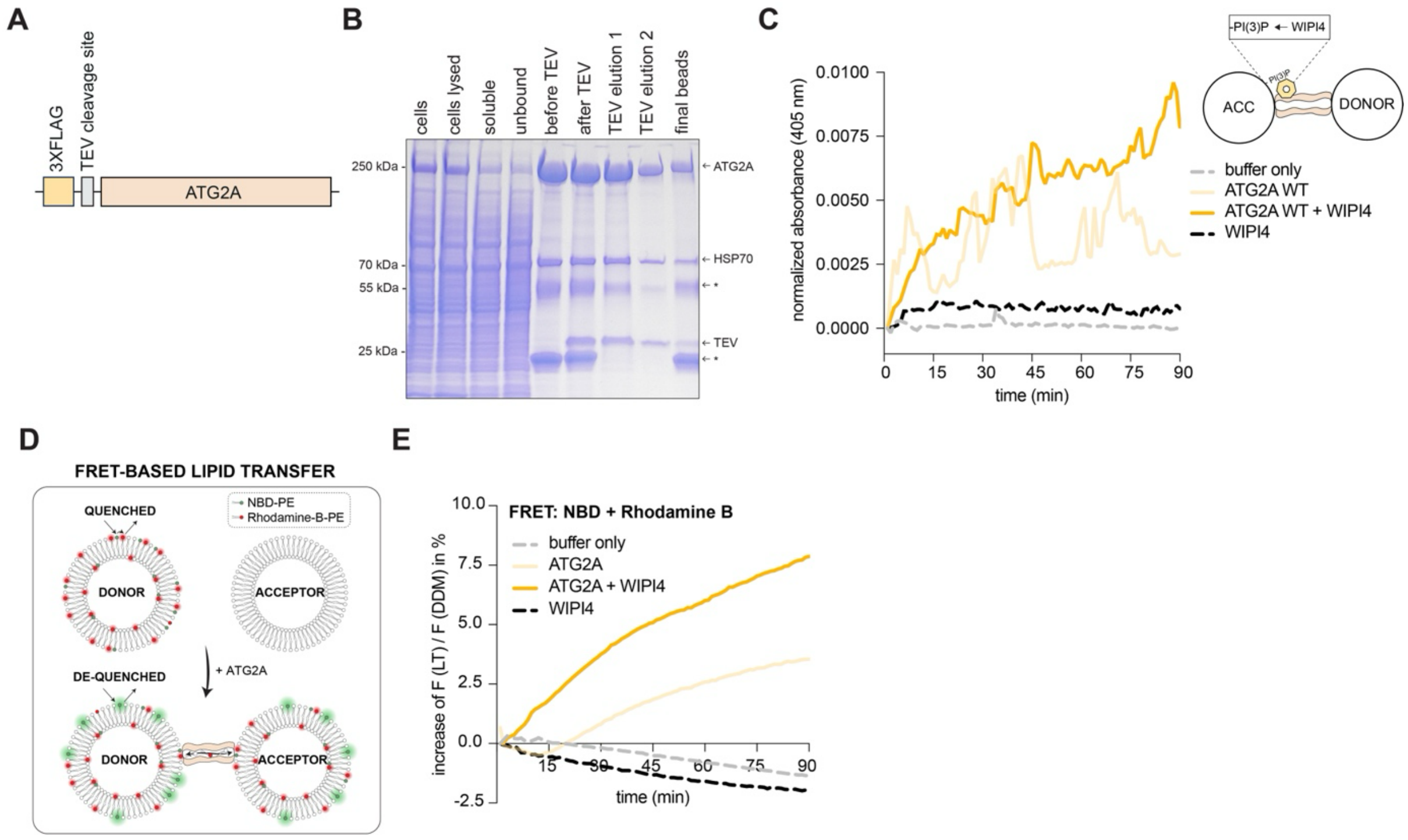


## Supplementary Fig. 4. Purification of ATG2A

(**A**) Schematic of expression construct used to produce ATG2A and ATG2A ΔLIR in HEK293T cells. A separate construct containing a C-terminal mCherry tag was employed in bead-based microscopy assays. (**B**) Coomassie-stained SDS-PAGE showing protein fractions from purification workflow. The fractions labeled “TEV elution 1” and “TEV elution 2” contain the cleaved and purified ATG2A protein. A common co-purifying contaminant, HSP70, and the TEV protease, are indicated on the gel. The two other bands derive from the anti-FLAG beads (**C**) Liposome tethering assay measured by absorbance at 405 nm. Lines represent mean values (*N* = 3; independent experiments). Conditions include: no protein (buffer only), 250 nM ATG2A wild-type (WT), and 250 nM ATG2A wild-type in presence of 100 nM WIPI4 or 100 nM WIPI4 alone. Data reflect increased turbidity upon liposome clustering. (**D**) Schematic of FRET-based lipid transfer assay using donor and acceptor SUVs. NBD fluorescence increases upon dilution of NBD-PE into acceptor membranes, indicating lipid transfer. (**E**) Comparison of lipid transfer activity between ATG2A WT (250 nM) in absence or presence of WIPI4 (100 nM), or WIPI4 alone (100 nM). NBD fluorescence (F(LT)) was recorded at 535 nm (excitation at 485 nm) and normalized to fluorescence after membrane solubilization with 0.5% DDM (F(DDM)). Acceptor SUVs contained 65% DOPC, 15% DOPS, 15% DOPE, and 5% PI3P whereas donor SUVs contained 66% DOPC, 30% DOPE, 2% NBD-DOPE, and 2% Rhodamine B-DOPE. All SUVs were extruded through a 100 nm filter. Experiments were performed in triplicate; lines represent mean values (*N* = 3; independent experiments).

**Supplementary Fig. 5**

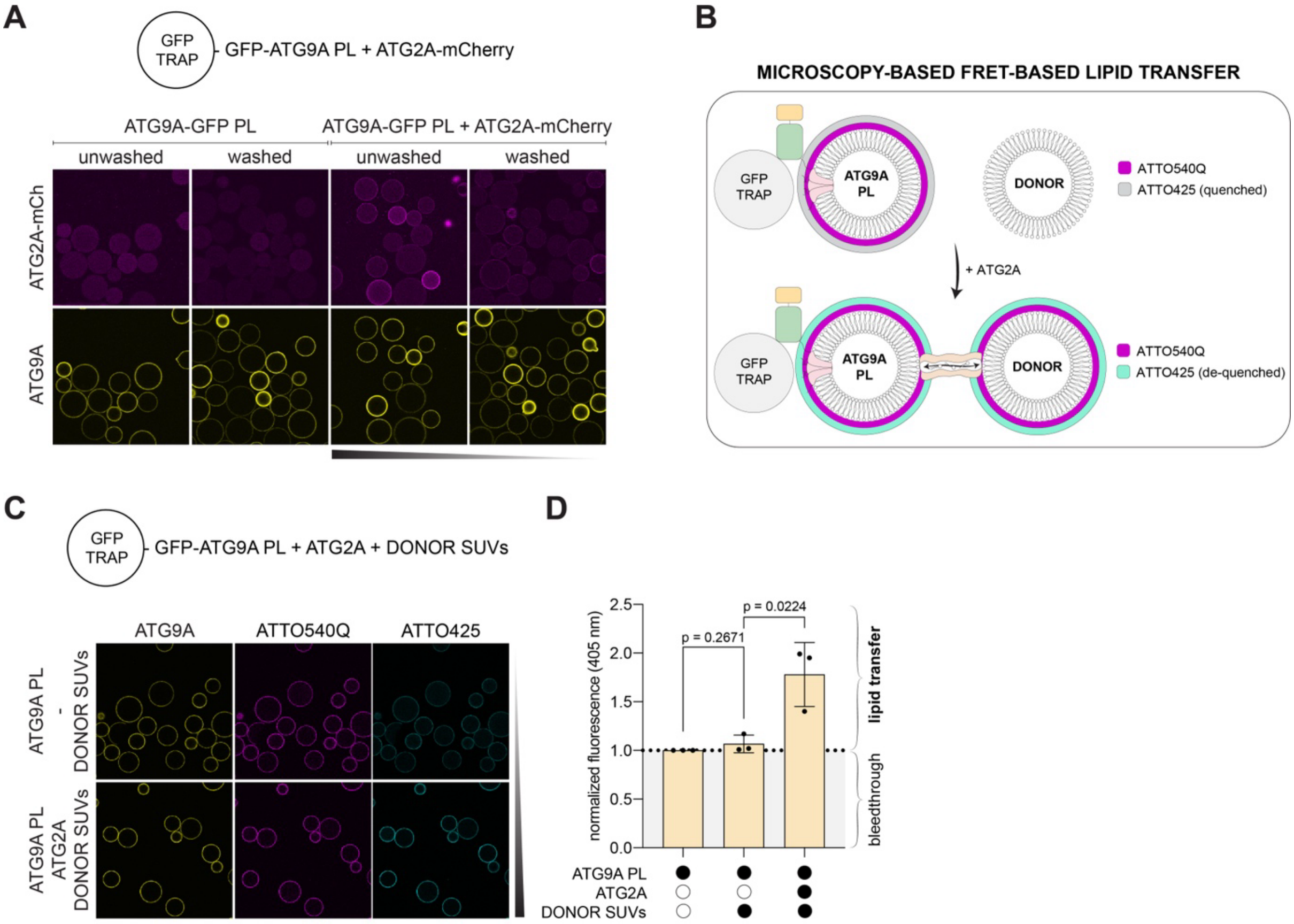


**Supplementary Fig. 5. Lipid transfer and tethering assays with ATG2A**

(**A**) ATG9A PL were incubated with ATG2A-mCherry for 2.5 hours, incubated with GFP-Trap agarose beads for 2.5 hours for immobilization of ATG9A PL and washed with high salt buffer. ATG9A PLs were composed of 50% POPE, 20% Cholesterol, 27% DOPC, 0% Liver PI, 2% DOPS, 1% Sphingomyelin (100 nm in size). (**B**) Schematic of the microscopy-based FRET-based lipid transfer assay using ATG9A PL including a ATTO425/ATTO540Q FRET pair and donor SUVs. ATTO425 fluorescence increases upon dilution of ATTO425-PE into donor membranes, indicating lipid transfer. (**C**) GFP-Trap agarose beads coated with ATG9A PL were incubated with donor SUVs (70% DOPC, 15% DOPE, 15% DOPS; 100 nm in size) in the presence or absence of ATG2A. The lipid composition of ATG9A PL reflected that of isolated ATG9A compartments supplemented with a ATTO425/ATTO540Q FRET pair (19% DOPC, 54% DOPE, 2% DOPS, 19% cholesterol, 1% sphingomyelin, 1% liver PI, 2% ATTO425-DOPE, 2% ATTO540Q-DOPE; 100 nm in size). (**D**) Quantification (*N* = 3; independent experiments) of ATTO425 fluorescence signal of the lipid transfer experiment shown in **C**. The fluorescence signal was normalized to the ATG9 PL-only control, which was set to 1 and is indicated by the dotted line. In this condition, the signal derived from the bleed-through of GFP containing ATG9A-GFP. Values above the line reflect ATTO425 dequenching

caused by increased separation from ATTO540Q, resulting from ATG2A-mediated lipid transfer. Statistical significance was assessed using an unpaired *t*-test. Bars represent mean ± s.d.

**Supplementary Fig. 6**

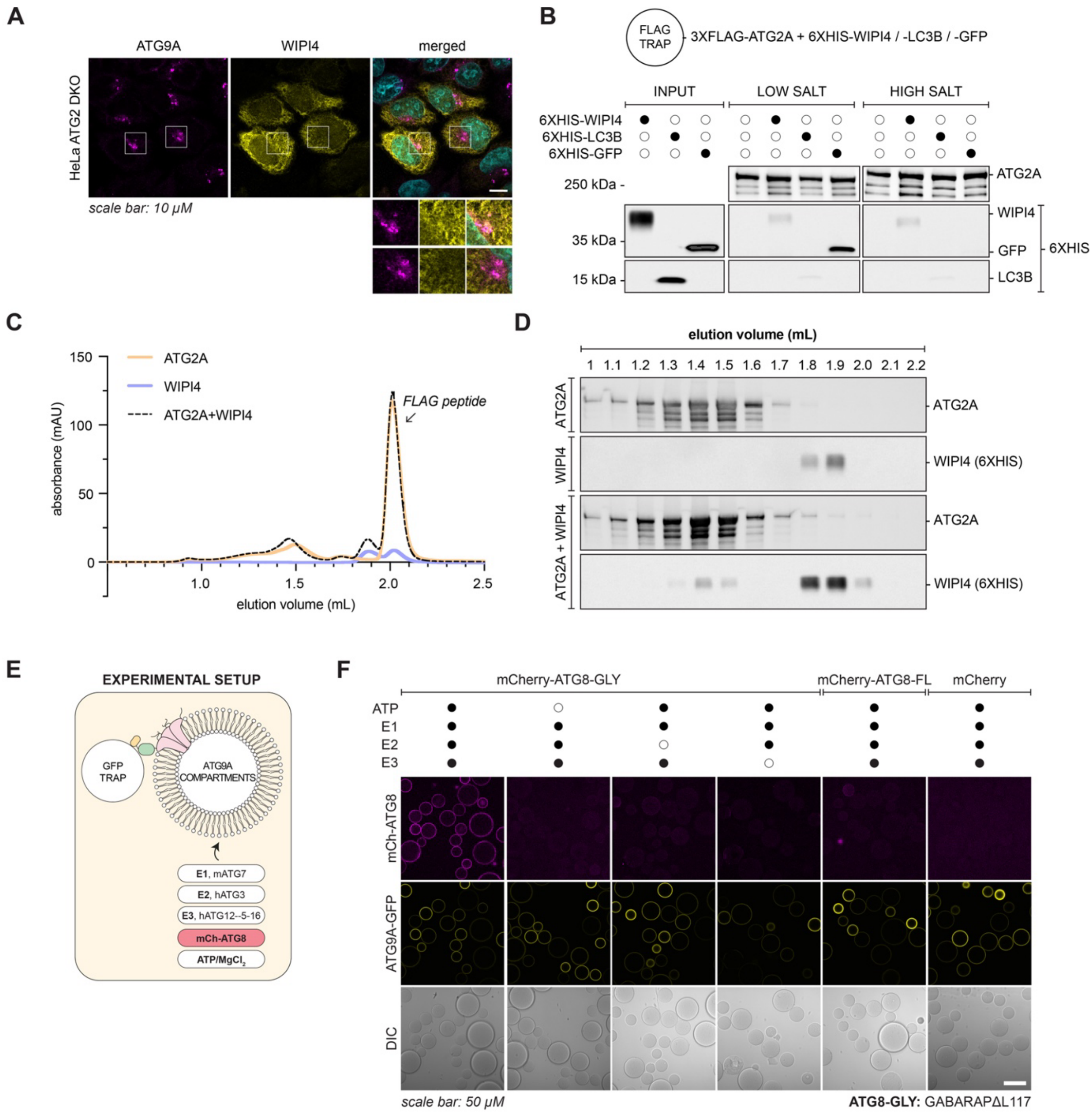


**Supplementary Fig. 6. ATG9A compartments can serve as substrates for ATG8 lipidation**

(**A**) Immunofluorescence staining of HeLa ATG2 DKO cells 24 h after transient transfection with EGFP-WIPI4. An antibody against EGFP was used to visualize WIPI4. Compared to other autophagy proteins (**Fig. S3A**), WIPI4 shows minimal localization to ATG9A-positive foci in ATG2 DKO cells, indicating that little or no PI3P is present at these sites. (**B**) FLAG Trap beads coated with 3×FLAG-ATG2A were incubated with 6×HIS-tagged WIPI4, LC3B, or GFP (negative control). After one wash with buffer containing 300 mM NaCl, signals were detected for all proteins (low-salt condition). Following additional washes, the GFP signal was lost, whereas WIPI4 and LC3B signals persisted (high-salt condition). Compared to the input,

signals of bound proteins were relatively weak. WIPI4, GFP, and LC3B were detected using an anti-HIS antibody. (**C**) Superose 6 size-exclusion chromatography (SEC) profiles of ATG2A, WIPI4, or a mixture of ATG2A (4 μM) and WIPI4 (4.8 μM). (**D**) Western blot analysis of SEC fractions from ATG2A, WIPI4, or ATG2A-WIPI4 mixtures. WIPI4 shows partial co-elution with the ATG2A peak, but most remains unbound. (**E**) Schematic of the experimental setup shown in **F**. (**F**) GFP-Trap agarose beads coated with native ATG9A-GFP compartments were incubated with mCherry-GABARAPΔL117 (GABARAP-GLY) in the presence of the ATG8 lipidation machinery: E1 (ATG7), E2 (ATG3), and E3 (a complex of ATG12–ATG5-ATG16L1), along with ATP, to assess lipidation. mCherry fluorescence on the bead surface indicates successful conjugation of GABARAP to ATG9A compartments. As negative controls, beads were incubated with either mCherry-GABARAP full-length (FL), which cannot be lipidated, or mCherry alone.

**Supplementary Fig. 7**

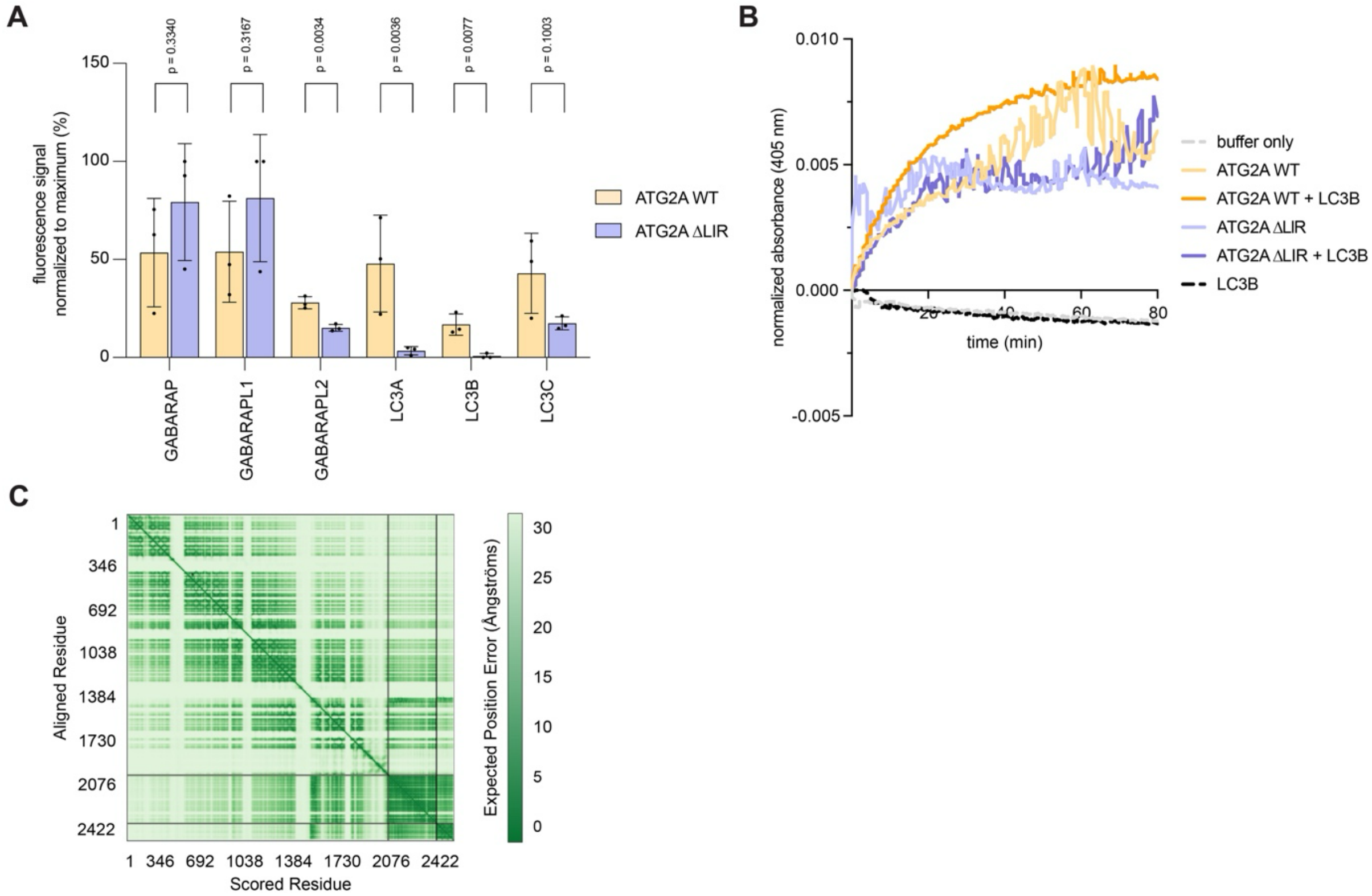


**Supplementary Fig. 7. ATG8 directly interacts with ATG2A and enhances its lipid transfer activity**

(**A**) Quantification of mCherry fluorescence signal from a microscopy-based bead assay assessing the recruitment of ATG2A to ATG8 proteins (as shown for LC3B in **Fig. 6A**). GSH-Trap beads coated with GST-tagged ATG8 family members (GABARAP, GABARAPL1, GABARAPL2, LC3A, LC3B, and LC3C) were incubated with either mCherry-tagged ATG2A or ATG2A-ΔLIR. Statistical significance was assessed using an unpaired *t*-test. Total fluorescence signals were normalized within each replicate to the respective maximum value. Each dot represents the mean of images acquired per replicate ($N$ = 3; independent experiments). Bars represent mean ± s.d. (**B**) Liposome tethering assay measured by absorbance at 405 nm. Lines represent mean values ($N$ = 4; independent experiments). Conditions include: no protein (buffer only), 250 nM ATG2A wild-type (WT) or mutant (ΔLIR), and 250 nM ATG2A wild-type or mutant (ΔLIR) in the presence of 100 nM 6XHIS-LC3B or 100 nM 6XHIS-LC3B alone. Data reflect increased turbidity upon liposome clustering, indicative of tethering activity (**C**) The predicted alignment error (PAE) heatmap for the AlphaFold3-predicted structure of the ATG2A-WIPI4-LC3B complex shown in **Fig. 6D**.
