# Supplementary material for: A lipid transfer-dependent feedback loop activates ATG9A compartments in autophagy initiation
Source: Nat Commun. 2026 Aug 31;17:10374. doi: 10.1038/s41467-026-77368-5 (PMC13627691; doi:10.1038/s41467-026-77368-5)
Supplement: Supplementary file 3 [file 41467_2026_77368_MOESM3_ESM.pdf]

nature portfolio

Peer Review File

A lipid transfer-dependent feedback loop activates ATG9A compartments in autophagy initiation

Corresponding Author: Professor Sascha Martens

This manuscript has been previously reviewed at another journal. This document only contains information relating to versions considered at Nature Communications.

This file contains all reviewer reports in order by version, followed by all author rebuttals in order by version.

Version 0:

Reviewer comments:

Reviewer #1

(Remarks to the Author)
The authors have carried out an excellent revision, and the improved Discussion now places the findings more clearly within a broader context. I still have a few important points, but I would consider them minor.

Response to point #1
I agree with the authors that some previous data regarding the membrane origin of autophagosomes may have been incorrectly interpreted because of CASM. This raises the possibility that this may also be, at least partially, the case in the authors' study, since ATG9A is not exclusively involved in autophagy. Indeed, pools of ATG9A have been shown to pass through, or function in, compartments where ATG8 lipidation can occur, such as the late Golgi or late endosomes. As an example for late endosomes, see PMID: 28877469. As a result, the authors cannot exclude the possibility that their omics analyses, and some of their in vitro analyses, contain a mixture of different ATG9A pools. Importantly, markers for these two compartments, late endosomes and the late Golgi, are missing from the analysis shown in Fig. S1E. Although it may be very difficult to resolve this experimentally, the authors should discuss this possible caveat for completeness. It would also be helpful if the analysis of late endosomal and Golgi markers could be included in Fig. S1E, to further substantiate the authors' argument that autophagy-dedicated ATG9A-positive compartments have been strongly enriched.
Regarding the citation of the study by Olivas et al. (2023), both here and in the response to my point #3, as well as in the relevant paragraph of the Discussion, I respectfully disagree with the authors' interpretation. To the best of my knowledge, this study did not exclude the possibility that the autophagosomal intermediate positive for ATG9A accumulated in the absence of the ATG2 proteins, is PI3P- and PI3KC3-positive.

Response to point #4
It is very unfortunate that the reported lipid-transfer-deficient ATG2 variants are not specific, but also display tethering defects. I may have missed it, but this finding should be mentioned explicitly in the manuscript. Although this is a negative result, I think the information would be very valuable for the community, particularly for researchers who use these ATG2 variants under the assumption that they are specifically lipid-transfer-deficient.

Reviewer #2

(Remarks to the Author)
None

Reviewer #3

(Remarks to the Author)
This manuscript was been modified to some extent based on my comments.
However, some points require furthe clarification. Some but not all are listed.
1. The authors do not address the issue that alternative models for isolation membrane (phagophore) formation exist. They

continue to adhere to the "prominent" model and do not address the possibility that the formation is from the ER. They should test their hypothesis with isolated ER vesicles.
2. In Figure 1 they have added a titration of Pi into the binding assay. This does not address the points raised.
In fact it causes further scurtiny of the results shown in 1G and 1H. It is not clear how are they normalizing the amount of ATG9A in the proteoliposomes with the amount in the ATG9Av? The authors should label the GFP-ATG9 beads to check the amount of ATG9A or run western blots to confirm amount for comparison.
Figure 1B- what condition are the cells incubated prior to vesicle isolation?
3. From this the analysis of contaminants in Fig. S1 is poorly done. First it is not clear why they used Beclin1. Secondly the amounts used and percentages loaded are not stated. It is quite easy to get a negative result from this approach.
4. t is unacceptable to ignore data from existing literature (Fesenko et al.) to avoid "ambiguity" regarding dataset selection.

Version 1:

Reviewer comments:

Reviewer #1

(Remarks to the Author)
The authors have satisfactorily addressed all my remaining minor comments.

Reviewer #3

(Remarks to the Author)
Thank you for your comments. I agree we disagree in the interpretation of the biology and but I am happy to accept the manuscript.

Open Access This Peer Review File is licensed under a Creative Commons Attribution 4.0 International License, which permits use, sharing, adaptation, distribution and reproduction in any medium or format, as long as you give appropriate credit to the original author(s) and the source, provide a link to the Creative Commons license, and indicate if changes were made.
In cases where reviewers are anonymous, credit should be given to 'Anonymous Referee' and the source.
The images or other third party material in this Peer Review File are included in the article’s Creative Commons license, unless indicated otherwise in a credit line to the material. If material is not included in the article’s Creative Commons license and your intended use is not permitted by statutory regulation or exceeds the permitted use, you will need to obtain permission directly from the copyright holder.
To view a copy of this license, visit https://creativecommons.org/licenses/by/4.0/

For your convenience, we have submitted an additional version of the manuscript file, in which all major points are highlighted in cyan.

Reviewer #1 (Remarks to the Author):

The authors have carried out an excellent revision, and the improved Discussion now places the findings more clearly within a broader context. I still have a few important points, but I would consider them minor.

We thank the reviewer for the positive assessment of our revised manuscript.

Response to point #1
I agree with the authors that some previous data regarding the membrane origin of autophagosomes may have been incorrectly interpreted because of CASM. This raises the possibility that this may also be, at least partially, the case in the authors' study, since ATG9A is not exclusively involved in autophagy. Indeed, pools of ATG9A have been shown to pass through, or function in, compartments where ATG8 lipidation can occur, such as the late Golgi or late endosomes. As an example for late endosomes, see PMID: 28877469. As a result, the authors cannot exclude the possibility that their omics analyses, and some of their in vitro analyses, contain a mixture of different ATG9A pools. Importantly, markers for these two compartments, late endosomes and the late Golgi, are missing from the analysis shown in Fig. S1E. Although it may be very difficult to resolve this experimentally, the authors should discuss this possible caveat for completeness. It would also be helpful if the analysis of late endosomal and Golgi markers could be included in Fig. S1E, to further substantiate the authors' argument that autophagy-dedicated ATG9A-positive compartments have been strongly enriched.

We thank the reviewer for raising this point. We agree that ATG9A localizes to multiple cellular compartments and that complete separation of distinct ATG9A pools remains technically challenging. To address this concern, we expanded the analysis presented in **Fig. S1E** (lines 106-110) and now include markers for the trans-Golgi network (TGN46) as well as late endosomal/lysosomal compartments (RAB7 and LAMP1), in addition to the previously shown markers. None of these proteins were enriched in the purified ATG9A vesicle fraction as detected by immunoblot analysis.

Regarding the citation of the study by Olivas et al. (2023), both here and in the response to my point #3, as well as in the relevant paragraph of the Discussion, I respectfully disagree with the authors' interpretation. To the best of my knowledge, this study did not exclude the possibility that the autophagosomal intermediate positive for ATG9A accumulated in the absence of the ATG2 proteins, is PI3P- and PI3KC3-positive.

We agree with the reviewer and have rephrased the statement accordingly in the revised manuscript (lines 303-308).

Response to point #4
It is very unfortunate that the reported lipid-transfer-deficient ATG2 variants are not specific, but also display tethering defects. I may have missed it, but this finding should be mentioned explicitly in the manuscript. Although this is a negative result, I think the information would be very valuable for the community, particularly for researchers who use these ATG2 variants under the assumption that they are specifically lipid-transfer-deficient.

We thank the reviewer for this comment and agree that this information may be valuable to the field. We have therefore explicitly included this observation in the revised manuscript (lines 261-265).

Reviewer #2 (Remarks to the Author):

None

Reviewer #3 (Remarks to the Author):

This manuscript was been modified to some extent based on my comments.
However, some points require furthe clarification. Some but not all are listed.

We thank the reviewer for taking the time to go through our revised manuscript. Below, we address the comments listed.

1. The authors do not address the issue that alternative models for isolation membrane (phagophore) formation exist. They continue to adhere to the "prominent" model and do not address the possibility that the formation is from the ER. They should test their hypothesis with isolated ER vesicles.

In the Introduction, we acknowledge that alternative models for phagophore formation have been proposed. Nevertheless, we maintain that substantial experimental evidence supports a model in which lipid transfer from the ER contributes to the formation and expansion of a vesicular precursor compartment containing

ATG9. To better reflect the current literature, we have expanded the Introduction by adding several references supporting this view (line 49), including:

https://pubmed.ncbi.nlm.nih.gov/33106658/
https://pubmed.ncbi.nlm.nih.gov/23549786/
https://pubmed.ncbi.nlm.nih.gov/22826123/
https://pubmed.ncbi.nlm.nih.gov/33439214/
https://pubmed.ncbi.nlm.nih.gov/29848619/
https://pubmed.ncbi.nlm.nih.gov/37115958/
https://pubmed.ncbi.nlm.nih.gov/37115157/

Taken together, these studies support the conclusion that a substantial fraction of lipids contributing to phagophore biogenesis are supplied by the ER.

In principle, the suggested experiments could be performed using microsomes isolated from cells. However, because such preparations inherently contain phospholipids including PI, PE, and PS, they would be expected to serve as substrates for PI3K-mediated phosphoinositide production and ATG8 lipidation in vitro. Consequently, these experiments would be unlikely to provide additional insight into the specific mechanistic questions addressed in the present study. We therefore consider them beyond the scope of this very extensive study.

2. In Figure 1 they have added a titration of Pi into the binding assay. This does not address the points raised.
In fact it causes further scurtiny of the results shown in 1G and 1H. It is not clear how are they normalizing the amount of ATG9A in the proteoliposomes with the amount in the ATG9Av? The authors should label the GFP-ATG9 beads to check the amount of ATG9A or run western blots to confirm amount for comparison.

We would like to draw the reviewer's attention to the second row of **Fig. 1G**, which shows the ATG9A-GFP signal and allows a comparison of the ATG9A amount in the different samples. To further address the reviewer's concern, we performed an additional western blot analysis comparing native ATG9A-containing vesicles and reconstituted ATG9A proteoliposomes. Analysis of the bound fractions revealed comparable amounts of ATG9A-GFP in the native vesicle and proteoliposome preparations (**Fig. S1J**, lines 1667-1675). Together, these data indicate that the differences observed in the PI3KC3-C1 binding assay are unlikely to be explained by substantial differences in the amount of ATG9A present in the samples.

Figure 1B- what condition are the cells incubated prior to vesicle isolation?

The vesicles were isolated from cells grown in rich medium, as stated now more clearly in the Methods section (line 727).

3. From this the analysis of contaminants in Fig. S1 is poorly done. First, it is not clear why they used Beclin1. Secondly the amounts used and percentages loaded are not stated. It is quite easy to get a negative result from this approach.

We used BECLIN1 as a control because it is also a subunit of the PI3KC3-C2 complex, which localizes to early endosomes. In the revised manuscript, we have expanded the panel of membrane markers to better assess potential contaminants, including TGN46, LAMP1, and RAB7 (**Fig. S1E**; lines 138-144 and 1648-1654). To directly address the concern regarding normalization and comparability of ATG9A amounts, we performed western blot analysis of input (0.5% of total sample loaded), bound (2.8% of total sample loaded), and eluted fractions (2.8% of total sample loaded) from ATG9A vesicle isolations performed in HAP1 wild-type, ATG9A-GFP, and ATG9A-GFP ATG2 DKO cells (as now indicated in the figure legends).

Importantly, the data show that ATG9A is strongly enriched in the eluted fraction, whereas the other marker proteins are not detectably present under the same conditions.

4. t is unacceptable to ignore data from existing literature (Fesenko et al.) to avoid "ambiguity" regarding dataset selectio

We are not ignoring the data from the Fesenko et al. paper. However, we have removed the direct comparison because the experimental systems are not directly comparable. Whereas Fesenko et al. isolated INVs, our study focuses on ATG9A-GFP-containing vesicles. We therefore consider a direct comparison of the respective datasets to be inappropriate. We apologize for including this comparison in the original version of the manuscript.
